# Comparison of Emergency Echocardiographic Results between Cardiologists and an Emergency Medicine Resident in Acute Coronary Syndrome 

**DOI:** 10.22037/aaem.v9i1.1247

**Published:** 2021-07-22

**Authors:** Fatemeh Rasooli, Farideh Bagheri, Azadeh Sadatnaseri, Haleh Ashraf, Maryam Bahreini

**Affiliations:** 1 Prehospital and Hospital Emergency Research Center, Tehran University of Medical Sciences, Tehran, Iran.; 2 Emergency Medicine Department, Tehran University of Medical Sciences, Tehran, Iran.; 3 Cardiology Department, Sina Hospital, Tehran University of Medical Sciences, Tehran, Iran.; 4 Emergency Medicine Department, Sina Hospital, Tehran University of Medical Sciences, Tehran, Iran.

**Keywords:** Emergency medicine, Cardiologists, Patient Discharge, Ultrasonography, Point-of-Care Systems

## Abstract

**Introduction:**

Early detection of regional wall motion abnormality (RWMA) can be a reliable tool for rapid disposition of patients with acute coronary syndrome (ACS) in the emergency department. In this study, the diagnostic accuracy of point-of-care echocardiography performed by a trained emergency medicine resident was evaluated in comparison with board-certified cardiologists.

**Methods:**

A prospective, cross-sectional study was implemented on adult patients with ACS. A trained emergency medicine (EM) PGY-3 resident performed point-of-care echocardiography under the supervision of two cardiologists and the reports were compared with cardiologists as a reference test.

**Results:**

100 patients with the mean age of 54.1 ± 11.5 years were recruited (65% male). Based on Thrombolysis in Myocardial Infarction (TIMI) and History, EKG, Age, Risk factors, and troponin (HEART) scores, 43.0% and 25.0% of patients were categorized as low-risk for ACS, respectively. The absolute measure of agreement between cardiologists to determine ejection fraction (EF) was 0.829 (95% CI: 0.74-0.89) based on intraclass correlation coefficient (ICC) estimation. The measurements of agreement between specialists and the EM resident based on the analysis of Kappa coefficient were 0.677 and 0.884 for RWMA and pericardial effusion, respectively. Moreover, 25 patients were in the-low risk group according to the HEART score with an agreement rate of 92% for the lack of RWMA between the EM resident and cardiologists.

**Conclusion:**

This study found acceptable agreement between the EM resident and cardiologists in assessing RWMA in different ACS risk groups. In addition, there was acceptable agreement between the EM resident and cardiologists in determining left ventricular ejection fraction (LVEF) and pericardial effusion.

## 1. Introduction:

It is crucial to manage the large number of patients who present to emergency departments (EDs) with acute chest pain with utmost accuracy.

Traditionally, medical history, physical examination, electrocardiography, and chest radiography have been used in the emergency ward for screening patients with cardiovascular complaints (1). However, these diagnostic tools are not completely accurate for exact disposition of those with acute coronary syndrome (ACS), especially in the low-risk group. 

Focused cardiac ultrasound (FOCUS) has become a vital tool in the evaluation of ACS patients (2), as suggested by the American Society of Echocardiography (ASE) and the American College of Emergency Physicians (ACEP) (3). It is a bedside, readily available, and noninvasive tool for real-time assessment of left ventricular ejection fraction (LVEF), intravascular volume, pericardial effusion, and assessment of regional wall motion abnormality (RWMA), as well as cardiac activity in patients with pulseless electrical activity (4-7). Echocardiography is considered a highly reliable modality to identify RWMA (8). Abnormal left ventricular wall motion can suggest a significant coronary artery obstruction. The sensitivity of transthoracic echocardiography alone for suspecting myocardial ischemia was found to be 91 percent for ACS disposition in low-risk patients. Also, early normal echocardiogram in suspected patients in the ED indicates a lower clinical risk (8).

Cardiologists routinely perform full standard transthoracic echocardiography (TTE) to manage ACS patients; however, they do not usually work full-time and are not always available in EDs. As a result of the delay in the disposition of ACS patients, emergency rooms become more crowded, which has financial consequences for both patients and hospitals. On the other hand, emergency medicine (EM) specialists constantly reside in EDs and are familiar with applying point-of-care ultrasound in emergency situations. The aim of this study was to assess the diagnostic accuracy of point-of-care echocardiography performed by a trained emergency medicine resident in comparison with board-certified cardiologists.

## 2. Methods:

***2.1 Study design*** ***and setting***

This is a prospective, cross-sectional study on adult patients (≥18 years-old) with acute coronary syndrome presenting to the EDs of two referral university hospitals with 50000-75000 annual visits from 2018 to 2019. The ethical aspects of this study were approved by Tehran University of Medical Sciences Institutional Review Board (Ethics code: IR.TUMS.VCR.REC.1398.144). Informed consent was taken from all patients after complete explanation of the study.

***2.2 Participants***

Patients with ST segment elevation myocardial infarction (MI) who needed primary coronary intervention (PCI) and participants with pacemaker implantation were excluded because of their influence on estimating cardiac wall motion. 

***2.3 Data gathering and procedure***

Data were recruited by an emergency medicine resident of postgraduate residency year (PGY)-3 as well as the cardiologists who were present in the hospitals to give cardiology consults in different days of the week in the morning and evening shifts.

According to the routine protocol, patients are emergently visited by emergency medicine residents, stabilized, and then visited by internal medicine residents by request for cardiology visits. Except for very low-risk ACS patients who are discharged, others stay in the emergency ward to undergo further testing for final disposition. Data were collected via convenient sampling. The EM resident initially spent a 2-hour theoretical course emphasizing on quantitative ejection fraction (EF) estimation, presence of pericardial effusion, and RWMA assessment, then passed a 20-hour hands-on training course under the supervision of the two cardiologists according to the emergency ultrasound guidelines of ACEP (1). 

The echocardiographs were performed using the ultrasound machines GE Vivid E9 and Samsung UGEO HM70A using phased-array 2-4 MHz transducers. Visual estimation was performed for the assessment of left ventricular function (LVF) both qualitatively and quantitatively, and focused ultrasound views were performed, including parasternal long axis, parasternal short axis, apical 4-chamber and subcostal views. The LVF according to the LVEF was categorized as normal (LVEF >55%), mild to moderate dysfunction (LVEF 35-55%), and severe dysfunction (LVEF35%) (9). Patients were assessed to determine whether they had RWMA or not. RWMA was assessed in four views including long axis, short axis, apical 4-chamber and two chamber views. Akinesia, hypokinesia and dyskinesia of left ventricle were considered as RWMA that can be detected immediately after an ischemic event preceding ECG and biomarker alternations (8). Besides, increased echogenicity, decreased thickness, and evidence of remodeling such as dyskinesia, as chronic ischemic changes, were evaluated. The sub-xiphoid view was also used to assess pericardial effusion. The study was performed in two phases. Echocardiography was first implemented by the emergency medicine resident, then by either cardiologist, who was blind to the resident’s results, at most 1 hour apart. Echocardiogram findings were documented along with other information such as History, EKG, Age, Risk factors, and troponin (HEART) and Thrombolysis in Myocardial Infarction (TIMI) scores in the questionnaire. 

The clinical decisions and interventions were performed according to the risk stratification of HEART score and based on the cardiologists’ decisions for moderate-risk patients. Patients with regional wall motion abnormality on FOCUS were admitted for further assessment. Acute coronary syndrome was defined as acute chest pain, dyspnea, or weakness, and also syncope with a cardiac cause.

***2.4 Outcomes***

First, the agreement between the EM resident and cardiologists was analyzed in assessment of cardiac function (RWMA, LVEF, and pericardial effusion) using point-of-care ultrasound in ACS. The examination results of the board-certified cardiologists were considered as gold standard. The results were also sub-analyzed based on the risk stratification of patients. 

***2.5 Statistical Analysis***

Based on a previous study, the kappa coefficient of agreement between EM specialists and cardiologists in evaluation of cardiac function was considered 0.71; with a confidence interval of 95% and a margin of error of 5%, a sample size of 88 patients was required (nQuery Advisor). Adding 10% to adjust for potential missing data, the final total sample size was about 100.

The mean differences of EF between the emergency medicine resident and the cardiologists were assessed using independent T-test. Furthermore, the Bland-Altman plot was used to measure the agreement between the two specialists. To construct a Bland-Altman plot, the difference between EF measured by specialists was plotted on the y-axis against the average of the total amount on the x-axis. Moreover, the intra-class correlation coefficient (ICC) was analyzed to assess the agreement on EF estimation. The specialists’ agreement on RWMA and pericardial effusion was assessed by measuring Kappa coefficient. In addition, the performance accuracy of the EM resident in determining RWMA was assessed in comparison with board-certified cardiologists as the gold-standard. Thus, we used Receiver Operating Characteristic (ROC) curve analysis and calculated sensitivity, specificity, and negative and positive predictive values with 95% confidence interval. The statistical uncertainty of calculated statistics was shown by 95% confidence interval. The level of significance was 0.05. Data were analyzed using Stata and Medcalc softwares. 

## 3. Results:

***3.1 Baseline characteristics of studied cases***

In this study, 100 patients with the mean age of 54.1 ± 11.5 (24-84) years were recruited (65.0% male). Of whom, 26.0% and 9.0% had a positive history of coronary angiography (CAG) and coronary artery bypass graft (CABG), respectively. Based-on the TIMI and HEART scores, 43.0% and 25.0% of patients were categorized as low-risk, respectively. Table 1 depicts the distribution of demographics, clinical presentations, vital signs, past history, and risk factors of patients at the time of admission.

***3.2 Echocardiographic findings***

***EF***

**Figure 1:**
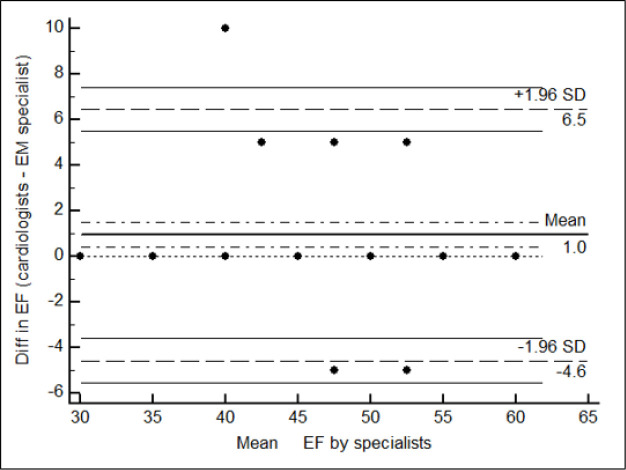
The Bland-Altman plot of ejection fraction (EF) which shows the agreement between the EM resident and cardiologists

The mean ejection fraction determined by cardiologists and the emergency medicine resident was 50.7 ± 4.8 vs. 49.8 ± 5.2 percent, respectively (P=0.001). Figure 1 presents the Bland-Altman plot of ejection fraction (EF) between the EM resident and cardiologists. The absolute measure of agreement between specialists for EF estimation was 0.829 (95% CI: 0.74-0.89) based-on intraclass correlation coefficient (ICC). We also analysed data after removing patients with a history of CAG or CABG. The mean EF determined by cardiologists in comparison with emergency medicine specialists was 51.9 ± 4.6 vs. 50.6 ± 5.2 percent, respectively (P=0.001). The absolute agreement of specialists for determining EF was 0.897 (95% CI: 0.80-0.94) based on intraclass correlation coefficient (ICC).

***3.3 RWMA and pericardial effusion***

The agreement rates between specialists and the EM resident based on analysing Kappa coefficient were 0.677 and 0.884 for RWMA and pericardial effusion, respectively. Furthermore, the performance of EM resident had the sensitivity and specificity of 82.9% and 86.2% for RWMA estimation and 80.0 and 100% for detecting pericardial effusion, respectively. 

From all low-risk patients according to the TIMI score (43 patients), 35 and 40 individuals were reported to have no RWMA by the EM resident and cardiologists on echocardiography, respectively. Thus, the agreement between them was 81.3%. Moreover, 25 patients were in the low-risk group according to the HEART score; of whom, 23 and 25 patients had normal wall motion according to the report of the EM resident and cardiologists, respectively. Therefore, the agreement between them was 92%. 

**3.4 Screening performance characteristics of echocardiography by EM resident**

Table 2, presents acuracy indices and predictive values in estimation of RWMA and pricardial effusion by the EM resident.

## 4. Discussion:

In our study, there was a moderate agreement between the EM resident and cardiologists with an acceptable accuracy. The specificity and negative predictive value of the echocardiograms of the EM resident improved when eliminating patients with a history of CAG or CABG. We found that the agreement on RWMA estimation was higher in low-risk patients according to the HEART score risk stratification in comparison with the TIMI score. Furthermore, the absolute agreement on EF estimation was acceptable. 

Emergency medicine specialists make critical decisions in a short time period, mandating goal-directed and focused point-of-care ultrasound in many circumstances. Regarding benefits and harms, the determination of EF and RWMA using FOCUS are important in low-risk patients with ACS to make more accurate dispositions and to reduce the possibility of unpredictable major cardiac events (1) in conjunction with risk stratification tools such as HEART/TIMI score. Considering the harmlessness of bedside ultrasound and the applicability of this module, it can be performed frequently in EDs. 

Previous studies have administered several training modules, which varied in duration and major outcomes. In Monsomboon's study, EM residents passed a 3-hour echocardiography training course focusing on LVEF visual estimation (10) and other researchers mentioned 4 to 6 hours of video programs (11-13). Furthermore, some aimed at training residents of EM or intensive care to get familiar with standard cardiac views within 3 to 12 hours (14, 15). In a study by Kerwin et al. trainees were capable of interpreting echocardiographic abnormalities with significant improvement after a 30-minute training period (16). Overall, these studies confirmed the trainees’ capabilities to perform echocardiography in comparison with cardiologists after the mentioned training time. 

**4.1. Assessment of RWMA**

The detection of RWMA by EM residents is very helpful in patients’ disposition. A small number of studies have evaluated the reliability of EM residents’ scans and few have assessed their agreement with cardiologists. The probability of coronary artery disease (CAD) increases with new RWMAs in patients with acute chest pain (17). On the other hand, the absence of RWMA in patients with ACS can lead to a safer discharge in conjunction with being categorized as low-risk based on a risk stratification tool. As mentioned in Arntfield's study, 98% of suspected CAD patients with no RWMA had a negative work-up for CAD (6). 

In our study, there was moderate agreement (0.677) between the EM resident and cardiologists with an accuracy of 85%. The specificity and negative predictive values of the echocardiograms by the EM resident were 86.2% and 90.3%, which improved by eliminating patients with history of CAG or CABG. Farsi et al. showed 92% agreement on RWMA between cardiologists and EM residents. Interestingly, the specificity and negative predictive value of echocardiograms performed by the EM resident were 87% and 98%, which are very close to our findings (13).

We found that the agreement on RWMA estimation was higher in patients who were low-risk according to the HEART score in comparison with the TIMI score. Thus, we propose using the combination of the HEART score with RWM status to speed up disposition of ACS patients from the emergency ward. In this regard, emergency specialists can more safely discharge low-risk patients with normal regional wall motion. 

**4.2. EF Assessment**

In this study, the mean EF measured by cardiologists was significantly higher than the EM resident (P=0.001), yet this difference was not considered clinically significant. The absolute agreement on EF estimation was 0.829 based on the interclass correlation coefficient (ICC), which presents an acceptable reliability. Eliminating patients with a history of CAG or CABG, the agreement reached 0.897, which may reflect better evaluation of LVEF by the EM resident in less complicated patients. In this regard, the agreement between the EM resident and the cardiologist on estimating ventricular function was 79.4% in Monsomboon's study (10). Also Moore and et al. showed weighted agreement of 84% between emergency physicians (EPs) and cardiologists with a weighted kappa of 0.61 (p < 0.001) in quantitative visual estimation of LVEF (12). In other studies, the agreement between trainees and cardiologists were considered acceptable with fair accuracy for LVEF estimation after minimal training (12, 14, 18-20). In a study by Bustam et al., the agreement between trainees and the cardiologist was 93% for visual estimation and 92.9 % for quantitative evaluation of LVEF and the Bland– Altman limits of agreement for LVEF assessment were similar to our study (11). This agreement was 91% in Farsi’s investigation (13).

**4.3. Assessment of Pericardial Effusion**

In this study, there was a desirable agreement between the EM resident and cardiologists in determining pericardial effusion, which is similar to Bustam's study with a sensitivity, speciﬁcity, PPV and NPV of 60, 100, 100 and 97.9 %, respectively (11). The accuracy of finding pericardial effusion by EM residents was 99% in Mandavia and Monsomboon’s studies (10, 21).

**Table 1:** Baseline characteristics of studied cases

Variable	Value
Age (year)	54.1 ± 11.5
Gender	
**Male**	65 (65.0)
**Female**	35 (35.0)
Vital signs	
**Systolic blood pressure (mmHg)**	136.2 ± 16.4
**Diastolic blood pressure (mmHg)**	81.6 ± 9.0
**Pulse Rate (bpm)**	75.3 ± 10.6
**Respiratory Rate (bpm)**	13.1 ± 1.6
**Saturation O2**	93.7 ± 2.5
Medical History	
**Angiography**	26 (26.0)
**CABG**	9 (9.0)
**ASA Use**	50 (50.0)
**Hypertension**	49 (49.0)
**Diabetes mellitus**	19 (19.0)
**Hyperlipidemia**	33 (33.0)
**Cigarette smoking**	40 (40.0)
Troponin I	
**Abnormal**	22 (22.0)
ECG changes	
**Not Visible**	8 (8.0)
**Not Noticeable**	59 (59.0)
**Clinically significant**	33 (33.0)
TIMI score	
**Low risk**	43 (43.0)
**Moderate risk**	47 (47.0)
**High risk**	10 (10.0)
HEART score	
**Low risk**	25 (25.0)
**Moderate risk**	54 (54.0)
**High risk**	21 (21.0)

Data are presented as mean ± standard deviation or number (%). SD: Standard deviation; CABG: Coronary Artery Bypass Graft; ASA: Acetylsalicylic Acid; ECG: Electrocardiogram; TIMI: Thrombolysis in Myocardial Infarction; HEART: History, EKG, Age, Risk factors, and troponin.

**Table 2:** Agreement and accuracy indices of regional wall motion abnormality (RWMA) and pricardial effusion between cardiologist (gold-standard) and emergency medicine (EM) resident

	Cardiologists			Kappa	Accuracy	Sesitivity	Spesificity	AUC	PPV	NPV
	**P**		**N**							
**RWMA in Total **										
EM resident	**P**	29	9	0.677	85.0%	82.9 (66.4,93.4)	86.2 (75.3,93.5)	0.85 (0.76,0.91)	76.3 (59.8,88.6)	90.3 (80.1,96.4)
	**N**	6	56							
**Pricardial Effusion**										
EM resident	**P**	4	0	0.884	99.0%	80.0 (28.4,99.5)	100 (96.2, 100)	0.90 (0.82,0.95)	100 (39.8, 100)	99.0 (94.3, 100)
	**N**	1	95							
**RWMA without** ** patients with prior angiography or ** **CABG**										
EM resident	**P**	15	7	0.682	85.0%	88.2 (63.6,98.5)	86.8 (74.7,94.5)	0.88 (0.77,0.94)	68.2 (45.1,86.1)	95.8 (85.7, 9.5)
	**N**	2	46							

Data are presented with 95% confidence interval. P: Positive; N: Negative in retest; CI: Confidence interval; AUC: Area Under the Curve; PPV: Positive predictive values; NPV: Negative predictive values; CABG: Coronary artery bypass graft.

## 5. Limitations and Suggestions

It is worth assessing the inter-rater reliability of cardiologists, which can be further studied although we had similar institutional protocols to carry out measurements. On the other hand, increasing the number of operators mandates coordination and this issue can be addressed in further studies to improve generalizability. Potential bias was reduced through cardiologists teaching echocardiographic measures. Differentiating between new and old RMWA is considered challenging even for skilled EM sonographers; thus, the EM resident in this study detected every abnormality without spending time on differentiation between the old and new lesions. Wall thickness measurements can differentiate old ischemia from a new one, which was not assessed in this study. On the other hand, the differential diagnoses of RWMA could confuse the operator, which are worth addressing. 

Applying a specific cardiac scoring system such as HEART score in conjunction with point-of-care echocardiography can lead to a faster and safer disposition focusing on RWMA in ACS patients. Patients who had a low risk of ACS according to HEART score with normal wall motion in echocardiography can have ambulatory follow-up for major cardiac events.

## 6. Conclusion:

This study found acceptable agreement between the EM resident and cardiologists in assessing RWMA, which was also seen in low-risk patients. Besides, there was acceptable agreement between the EM resident and cardiologists in determining LVEF and pericardial effusion. 

## 7. Declarations:

### 7.1 Acknowledgement

We kindly thank Dr. Mehdi Mehrani, MD for his valuable cooperation in carrying out this study. 

### 7.2 Funding

None

### 7.3 Author Contributions

Concept and design: FR, FB, MB, AS, HA

Data collection: FB, AS, HA

Writing the article: FR, MB, FB, AS, HA

Critical revision of the article: FR, MB, AS, HA, FB

Final approval of the article: FR, MB, AS, HA, FB

### 7.4 Conflict of Interest

None
